# EARLY DIAGNOSIS OF SEVERE COMBINED IMMUNODEFICIENCIES

**DOI:** 10.1590/1984-0462/;2017;35;1;00017

**Published:** 2017

**Authors:** Luiz Vicente Rizzo

**Affiliations:** a Diretor de Pesquisa, Hospital Israelita Albert Einstein, São Paulo, SP, Brasil.

Severe combined immunodeficiencies (SCID) are a group of hereditary diseases mainly characterized by T-cell lymphopenia. These diseases are recognized as pediatric emergencies due to their high morbidity and mortality. Data suggest that more than 90% of children with an early diagnosis of SCID who are able to receive a bone marrow transplant before the age of four survive. Of those receiving treatment after four years of age, only 50% survive. This data indicate that early diagnosis is critical.

Neonatal screening done by quantifying T-cell receptor excision circles (TRECs) allows for the diagnosis of severe immunodeficiencies in the immediate postnatal period. The sensitivity of neonatal diagnosis of typical severe combined immunodeficiencies using the TREC count is around 100% and depends on the establishment of a baseline for the minimum number of TRECs considered normal in subjects without severe combined immunodeficiency. Adapting an algorithm for preterm infants/children with signs and symptoms of immunodeficiency greatly reduces the number of false positives. By adding on the evaluation of the kappa-deleting recombination excision circles (KRECs), which are present in cell differentiation, there is a gain in the diagnosis of some B-lymphocyte deficiencies, that have a greater incidence than the severe combined immunodeficiencies.

The study presented in this issue of the *Revista Paulista de Pediatria*, “Neonatal Screening of Severe Combined Immunodeficiencies by TRECs and KRECs: Second Pilot Study in Brazil,” by Kanegae, Barreiros et al.,^1^ demonstrates that screening will be useful in Brazil. It is worth noting that Brazil is a country where compulsory and necessary BCG vaccine adds a challenge to the management of these children. Witnessing the development of these technologies in the country at the service of the population is auspicious. The work to reduce the cost of this type of cutting-edge technology must be continuous, and considering that it is really useful, it should guide the advances so that the examinations can be readily available on a large scale.
